# Contribution of microRNA to pathological fibrosis in cardio-renal syndrome: impact of uremic toxins

**DOI:** 10.14814/phy2.12371

**Published:** 2015-04-20

**Authors:** Indrajeetsinh Rana, Andrew R Kompa, Joanna Skommer, Bing H Wang, Suree Lekawanvijit, Darren J Kelly, Henry Krum, Fadi J Charchar

**Affiliations:** 1 School of Health Sciences, Federation University AustraliaBallarat, Victoria, Australia; 2 Centre of Cardiovascular Research and Education in Therapeutics Monash UniversityMelbourne, Victoria, Australia; 3 Department of Medicine, University of Melbourne, St. Vincent's HospitalFitzroy, Victoria, Australia

**Keywords:** AST-120, indoxyl sulfate, microRNA 21, microRNA 29b, myocardial infarction, uremic toxin

## Abstract

Progressive reduction in kidney function in patients following myocardial infarction (MI) is associated with an increase in circulating uremic toxins levels leading to increased extracellular matrix deposition. We have recently reported that treatment with uremic toxin adsorbent AST-120 in rats with MI inhibits serum levels of uremic toxin indoxyl sulfate (IS) and downregulates expression of cardiac profibrotic cytokine transforming growth factor beta (TGF-*β*1). In this study, we examined the effect of uremic toxins post-MI on cardiac microRNA-21 and microRNA-29b expression, and also the regulation of target genes and matrix remodeling proteins involved in TGF*β*1 and angiotensin II signaling pathways. Sixteen weeks after MI, cardiac tissues were assessed for pathological and molecular changes. The percentage area of cardiac fibrosis was 4.67 ± 0.17 in vehicle-treated MI, 2.9 ± 0.26 in sham, and 3.32 ± 0.38 in AST-120-treated MI, group of rats. Compared to sham group, we found a twofold increase in the cardiac expression of microRNA-21 and 0.5-fold decrease in microRNA-29b in heart tissue from vehicle-treated MI. Treatment with AST-120 lowered serum IS levels and attenuated both, cardiac fibrosis and changes in expression of these microRNAs observed after MI. We also found increased mRNA expression of angiotensin-converting enzyme (ACE) and angiotensin receptor 1a (Agtr1a) in cardiac tissue collected from MI rats. Treatment with AST-120 attenuated both, expression of ACE and Agtr1a mRNA. Exposure of rat cardiac fibroblasts to IS upregulated angiotensin II signaling and altered the expression of both microRNA-21 and microRNA-29b. These results collectively suggest a clear role of IS in altering microRNA-21 and microRNA-29b in MI heart, via a mechanism involving angiotensin signaling pathway, which leads to cardiac fibrosis.

## Introduction

Kidney dysfunction is a common consequence of myocardial infarction (MI), and results in a greater risk of in-hospital death and cardiovascular events such as heart failure and stroke (Anavekar et al. 2004; Goldberg et al. 2005, 2009; Parikh et al. 2008). We have recently demonstrated that elevated serum uremic toxins levels, such as indoxyl sulfate (IS), lead to a progressive decline in kidney function as well as increased levels of cardiac TGF-*β*1 and collagen protein in rat model of MI (Lekawanvijit et al. 2013). Indoxyl sulfate is a small nondialyzable protein-bound uremic toxin derived from dietary tryptophan. It is metabolized by the gut microflora to the precursor indole where it is absorbed across the gastrointestinal tract prior to its conjugation to IS by the liver (Lekawanvijit et al. 2012b). AST-120 is an inert carbon-based oral adsorbent that has been used clinically to bind indole in the gut preventing its absorption into the circulation (Aoyama and Niwa 2001). Lowering uremic toxin levels in MI rats using the oral adsorbent AST-120, reduced cardiac expression of collagen I protein and transforming growth factor (TGF-*β*1) (Lekawanvijit et al. 2013). However, the molecular mechanisms involved in post-MI IS-mediated upregulation of TGF-*β*1 and fibrosis is not known.

MicroRNAs are small endogenously transcribed regulatory RNA that modulates gene expression by binding to either 3′ or 5′ untranslated regions (UTR) of mRNA or promoter sequences. These master regulators of cell homeostasis are known to regulate many cardiac processes and play a role in cardiac dysfunction (Thum et al. 2008; Qin et al. 2011; Salic and De Windt 2012). MicroRNA-21 and microRNA-29 are among the most abundantly expressed microRNAs in heart and are known to regulate fibrosis by their action on mRNA of extracellular matrix proteins and TGF-*β*1 (van Rooij et al. 2008; Liang et al. 2012). MicroRNA-21 has an inhibitory effect on sprouty 1 (Spry1), phosphatase and tensin homolog (PTEN) and Smad 7, all known to suppress the TGF-*β*1 signaling pathway (Zhang et al. 2007; Thum et al. 2008; Roy et al. 2009; Adam et al. 2012; Li et al. 2013), therefore increases in microRNA-21 may lead to an upregulation of TGF-*β*1 signaling. TGF-*β*1, collagen 1A1 and fibronectin-1 are direct targets of microRNA-29b, hence an increase in microRNA-29b may lead to inhibition of TGF-*β*1 signaling (Wang et al. 2012; Xiao et al. 2012; Zhang et al. 2014).

Angiotensin II has been shown to activate Smad pathways via TGF-*β*-dependent (Sorescu 2006; Huang et al. 2010) and -independent mechanisms (Rodriguez-Vita et al. 2005; Rodrigues Diez et al. 2010). Treatment of cardiac fibroblast cells with angiotensin II increased expression of TGF-*β*1, microRNA-21 but inhibited expression of microRNA-29b (Zhang et al. 2014). Earlier studies have reported involvement of the angiotensin type 1 receptor (Agtr1) in angiotensin II-mediated upregulation of TGF-*β*_1_ expression in cardiac myocytes and fibroblasts (Rosenkranz 2004). However, the effect of IS on cardiac angiotensin II signaling in the MI heart is not known. Also, an important question, whether the uremic toxin IS promotes myocardial fibrosis by altering cardiac TGF*β*1-microRNA-21 and/or TGF*β*1-microRNA-29b signaling remains unexplored.

This study is the first to examine the role of uremic toxins post-MI on cardiac microRNA-21 and microRNA-29b expression, and examine the regulation of target genes and matrix remodeling proteins involved in TGF*β*1 and angiotensin II signaling pathways. In vitro studies using cardiac fibroblast cells will confirm the direct effects of IS on these microRNAs and target gene expression.

## Materials and Methods

### Animals

Male Sprague–Dawley rats (220–250 g) underwent left anterior descending (LAD) ligation to induce myocardial infarction (MI) by methods described earlier (Rana et al. 2010; Dworak et al. 2012; Kompa et al. 2013). Briefly, animals were intubated and artificially ventilated with 2% isoflurane in oxygen. A left thoracotomy was performed and the LAD coronary artery ligated with a 6–0 prolene suture a 2–3 mm below its origin. Visible blanching and hypokinesis of the anterior LV wall were indicative of successful ligation. A sham operation involved the same procedure except the LAD was not ligated. The thorax was then closed after briefly inflating the lungs to expel air from the thoracic cavity, and the skin was sutured. On recovery, MI animals were randomized to receive either AST-120 (MI+AST-120, *n* = 9) or no treatment (MI+vehicle, *n* = 8) for 16 weeks. AST-120 (Kremezin®, Kureha Pharmaceuticals, Tokyo, Japan) was administrated postoperatively in the chow at 8% w/w with unrestricted access. Sham-operated rats (*n* = 12) were used as controls.

Serum IS levels were measured using high-performance liquid chromatography method (Shimadzu, Kyoto, Japan) at study endpoint (16 weeks post-MI). In the final week of the respective study, echocardiography performed for assessment of cardiac function, and blood pressure measurements recorded prior to tissue harvest. Tissues were assessed for changes in gene expression using real-time PCR. The investigation conformed to the Guide for the Care and Use of Laboratory Animals published by the US National Institutes of Health (PHS Approved Animal Welfare Assurance No. A5587-01). All animal usage was also approved by the St Vincent's Hospital's Animal Ethics Committee (AEC) in accordance with the National Health and Medical Research Council (NHMRC) guide for the care and use of laboratory animals.

### Cardiac function assessment – echocardiography and hemodynamic measurements

Echocardiography was performed in lightly anesthetized animals (ketamine 40 mg/kg, xylazine 5 mg/kg, i.p.) using a Vivid 7 (GE Vingmed, Horten, Norway) echocardiography machine with a 10 MHz phased array probe. The procedure was performed as per published protocol routinely used in our laboratory (Rana et al. 2010; Kompa et al. 2013). For hemodynamic assessment, animals were intubated, and under positive pressure ventilation, a micromanometer (Millar Instruments, Houston TX) was inserted into the right common carotid artery for measuring blood pressure and heart rate, recorded on a Powerlab using Chart (v5) software (ADInstruments, Bella Vista, NSW, Australia).

### Glomerular filtration rate (GFR)

One day prior to sacrifice, GFR was performed to measure kidney function. Briefly, animals were injected with 0.26 mL (i.v.) of the radioactive isotope, ^99^technetium-diethylene triamine penta-acetic acid (^99^Tc-DTPA) prepared at a rate of 37 MBq/ml (1 mCi/mL). Animals were bled 43 min later and their plasma radioactivity measured and compared to the counts of the standard reference prepared at the time of injection. GFR/kg body weight was calculated as described in previous studies published by our group (Kelly et al. 1998; Lekawanvijit et al. 2012a).

### Neonatal rat cardiac fibroblast culture and treatment

We used isolated neonatal rat cardiac fibroblasts (NCF) in our study to investigate direct effects of indoxyl sulfate on cardiac cells on expression genes and microRNAs involved in cardiac fibrosis during cardiac remodeling. NCF were isolated from 1- to 2-day-old pups with enzymatic digestion as described in detail previously (Simpson 1985; Woodcock et al. 2002). All animal usage was also approved by AMREP Animal Ethics Committee (AEC) in accordance with the National Health and Medical Research Council (NHMRC) guide for the care and use of laboratory animals (E/0980/2010/M). NCF were seeded and maintained in high-glucose (25 mmol/L) DMEM (Invitrogen, Mount Waverley, Vic, Australia) in the presence of 1% antibiotic/antimycotic (Invitrogen) and 10% FBS (JRH biosciences). NCF were used at passage 2 (See et al. 2004; Lekawanvijit et al. 2010). NCF were then seeded at a density of 250,000 cells/well in 6-well plates and incubated (5% CO_2_) overnight before serum starved with 0.5% bovine serum albumin (BSA) for 48 h. Cells were then treated with 10 *μ*mol/L indoxyl sulfate in the presence of 0.5% BSA in DMEM/F12 media. After 6 and 18 h of further incubation, cells were harvested and total RNA was extracted using Ambion® RNAqueous kits (AM1912, Ambion, Life Technologies Australia Pty Ltd, Mulgrave, Vic., Australia). Three independent experiments were performed with triplicate samples in each group.

### RNA extraction

RNA was extracted using Qiagen RNeasy kits (Qiagen, Hilden, Germany) from the viable myocardium of the remote noninfarct region of the left ventricle (30 mg) from MI rats. This region of the myocardium was chosen because it receives even exposure to uremic toxins concentrations observed in the circulation. A similar region of the heart was obtained for sham rats (Lekawanvijit et al. 2013). Then cDNA synthesis was performed using microRNA-specific primers (Applied Biosystems, Foster City, CA), by method provided by the manufacturer. Standard cDNA synthesis protocol was followed to obtain cDNA for gene assays (Nankervis et al. 2013).

### Quantitative PCR for microRNAs

Quantitative PCR experiments were performed using TaqMan assays (Applied Biosystems) by method described previously (Szafranska et al. 2008). In brief, we added 10 ng total RNA, carried out reverse transcription in duplicate (16°C for 30 min, 42°C for 30 min, 85°C for 5 min, and then to 4°C), and then conducted PCRs in duplicate from each reverse transcription reaction (95°C for 1 min and 40 cycles of 95°C for 15 sec and 60 °C for 30 sec). All PCR amplifications were performed on ViiA 7 Real-Time PCR system (Applied Biosystems) using ViiA 7 RUO Software. mRNA was normalized to U87 endogenous control and the relative fold difference in expression was calculated using the 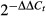 method (Wilkinson-Berka et al. 2009).

### Quantitative PCR for genes

Quantitative PCR experiments were performed using TaqMan assays (Applied Biosystems) by method described previously (Nankervis et al. 2013). mRNA was normalized to GAPDH endogenous control and the relative fold difference in expression was calculated using the 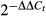 method (Wilkinson-Berka et al. 2009). All PCR amplifications were performed on ViiA 7 Real-Time PCR system (Applied Biosystems) using ViiA 7 RUO Software. The primer sequences used in the study are found in Table1.

**Table 1:** Cardiac and renal measurements from Sham, MI+Vehicle and MI+AST-120-treated animals. Data presented as mean ± SEM.

	Sham (*n* = 12)	MI+Veh (*n* = 8)	MI+AST-120 (*n* = 9)
Kidney data			
Renal Interstitial Fibrosis (% area)	1.92 ± 0.15	4.22 ± 0.14***	3.32 ± 0.18***^,^##
Kim-1 Positive Renal Tubule	11.00 ± 2.40	25.25 ± 7.11*	6.56 ± 1.97##
Glomerular Filtration Rate (mL/min/kg)	10.64 ± 0.36	9.88 ± 0.41	9.51 ± 0.43
Indoxyl Sulfate Data			
Serum Indoxyl Sulfate Levels 8 weeks (mg/dL)	0.18 ± 0.02	0.21 ± 0.01	0.04 ± 0.01***^,^###
Serum Indoxyl Sulfate Levels 16 weeks (mg/dL)	0.18 ± 0.02	0.25 ± 0.03*	0.04 ± 0.01***^,^###
Delta Serum Indoxyl Sulfate (IS) (mg/dL)	0.07 ± 0.02	0.15 ± 0.04*	−0.10 ± 0.02***^,^###
Cardiac Data			
Systolic Blood Pressure (mmHg)	90.68 ± 3.34	89.26 ± 4.43	86.07 ± 3.19
Diastolic Blood Pressure (mmHg)	62.36 ± 3.11	63.84 ± 4.92	62.56 ± 3.08
Heart Rate (BPM)	275.75 ± 14.28	279.38 ± 9.78	292.44 ± 17.23
Preload Recruitable Stroke Work (mmHg)	83.67 ± 6.76	58.62 ± 4.76**	69.35 ± 6.55
Tau Logistic (msec)	9.64 ± 0.29	13.42 ± 0.93**	11.87 ± 0.79*
Tau Weiss (msec)	12.87 ± 0.38	16.97 ± 0.87**	15.56 ± 0.97*
LV End Diastolic Pressure (mmHg)	4.48 ± 0.31	6.65 ± 0.84*	6.53 ± 0.57*
dP/dt max (mmHg/sec)	5636 ± 163	4653 ± 208**	4647 ± 182**
dP/dt min (mmHg/sec)	−5252 ± 231	−3458 ± 179***	−3442 ± 193***
Collagen I/Actin (Relative Protein expression)	0.10 ± 0.02	0.63 ± 0.09***	0.11 ± 0.06###
TGF-*β*1 mRNA (Fold Change Relative to Sham)	1.00 ± 0.04	1.37 ± 0.11**	0.91 ± 0.072###
Cardiac Infarct Size (%)	0.0	41.63 ± 3.47***	44.61 ± 52.89***
Fractional Shortening (%)	39.29 ± 1.71	24.71 ± 3.78**	20.40 ± 2.54**
Left Ventricular Ejection Fraction (%)	66.41 ± 1.82	40.31 ± 5.22**	42.11 ± 4.48**

^*^P < 0.05, ^*^^*^P < 0.01, ^*^^*^^*^P < 0.001 versus Sham. ^#^*P* < 0.05, ^##^*P* < 0.01, ^###^*P* < 0.001 versus MI+Vehicle.

Data analyzed by performing one-way ANOVA followed by Bonferroni post hoc analysis.

### Western blot analysis

Noninfarcted myocardial tissue (30 mg) was homogenized with 1 mL of modified RIPA buffer in the presence of protease and phosphatase inhibitors. Equal amounts of protein (30 *μ*g) were separated by 10% sodium dodecyl sulfate-polyacrylamide gel electrophoresis, and electrophoretically transferred to nitrocellulose membranes (Amersham Biosciences). Western blot analysis was performed as per manufacturer's protocol with collagen- I (Novus Biologicals, Littleton, CO) and pan-actin antibody (NeoMarkers, Fremont, CA) and then visualized by enhanced chemiluminescence (Thermo Scientific, Rockford, IL). Band intensity was analyzed using ImageJ software (U.S. National Institutes of Health, Bethesda, MD). Pan-actin antibody was used to measure endogenous controls proteins.

### Quantitation of fibrosis

Four micron paraffin-embedded tissue sections from the heart and kidney were stained with picrosirius red to determine the level of fibrosis in sham and MI rats. Ten random nonoverlapping fields from the remote noninfarct cardiac regions and the renal cortex-to-corticomedullary region (glomeruli excluded) of all animals were captured using a microscope attached to a digital camera (Carl Zeiss AxioVision, Germany). Fibrosis was evaluated by calculating the proportional area of picrosirius red-stained matrix using image analysis (AIS, Analytical imaging Station Version 6.0, Imaging Research Inc, Ontario, Canada).

### Kidney injury molecule-1 (KIM-1) expression

Tissue expression of kidney KIM-1 was assessed by immunohistochemistry (Lekawanvijit et al. 2013), using goat antiKIM-1 (R&D systems, Minneapolis, MN, 1:200 dilution) antibody. Numbers of KIM-1-positive tubules were counted from whole kidney sections under a microscope.

### Statistical analysis

Data are presented as mean ± SEM. One-way ANOVA with Bonferroni's multiple comparison test or Kruskal–Wallis test with Dunn's multiple comparison test were used for comparisons among all groups for parametric and nonparametric data, respectively. For comparisons between two groups, unpaired Student's *t*-test was used for parametric data and Mann–Whitney test for nonparametric data. All statistical analyses were performed using GraphPad Prism 5 (Version 6.04, La Jolla, CA). The presence of correlations between variables was analyzed using Pearson's correlation analysis.

## Results

### Serum IS levels

Compared to sham animals, the serum IS level at 16 weeks was 38% higher in MI+Vehicle-treated rats (*P* < 0.05, Table1). Serum IS level in AST-treated MI rats was significantly lower compared to both sham and MI+Vehicle rats (*P* < 0.001 compared to MI+Veh, Table1). Compared to sham animals, the difference in serum IS levels before and after treatment at 16 weeks, (Delta IS) were 2.5-fold higher in MI+Vehicle rats (*P* < 0.05, Table1). This was completely attenuated by AST-120 treatment (*P* < 0.001 compared to MI+Veh, Table1).

### Cardio-renal fibrosis and cardiac function data

Compared to sham animals, the percentage of renal fibrosis was increased 2.2-fold in vehicle-treated MI animals (*P* < 0.001, Table1). In AST-120-treated MI animals, the percentage of renal fibrosis was significantly attenuated compared to the vehicle MI group (*P* < 0.01) but was higher compared to sham animals (*P* < 0.001, Table1). Compared to sham animals, expression of the renal injury marker Kim-1 was 2.29-fold higher in vehicle-treated MI animals (*P* < 0.05, Table1). This increase in Kim-1 expression was attenuated by AST-120 treatment (*P* < 0.01 compared to MI+Veh, Table1). There was a significant reduction in the percentage of left ventricular fractional shortening and left ventricular ejection fraction in vehicle-treated MI animals compared to sham animals (*P* < 0.01). Reduction in the percentage of left ventricular fractional shortening after MI was not different in AST-120 treatment group compared to MI+Veh group of animals (*P* = 0.351, Table1). Left ventricular infarct size was similar in both MI groups (*P* < 0.001 compared to sham for both, Table1).

**Figure 1:**
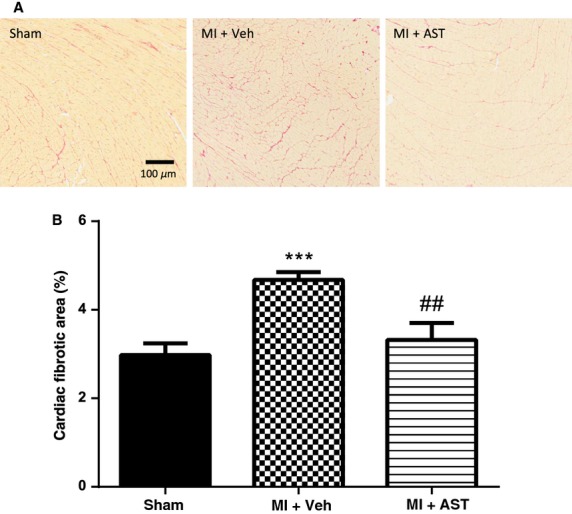
(A) Figure shows picrosirius red staining in heart from sham, MI+Vehicle and Mi+AST group of rats (B) Quantitative analysis of the percentage area of cardiac fibrosis measured by picrosirius red staining. Data presented as mean±SEM. ***P < 0.001 versus Sham. ^##^*P* < 0.01 versus MI+Vehicle. Data analyzed by performing a one-way ANOVA followed by Bonferroni post hoc tests.

Picrosirius red staining in the heart was evident in all three groups (Fig.1A) but it was highest in vehicle-treated MI group. The percentage area of cardiac fibrosis was significantly higher in vehicle MI group compared to sham animals (*P* < 0.001, Fig.1B). The AST-120-treated MI group had significantly reduced cardiac fibrosis compared to the vehicle MI group (*P* < 0.01, Fig.1B).

### Cardiac microRNAs

#### microRNA-21

**Figure 2:**
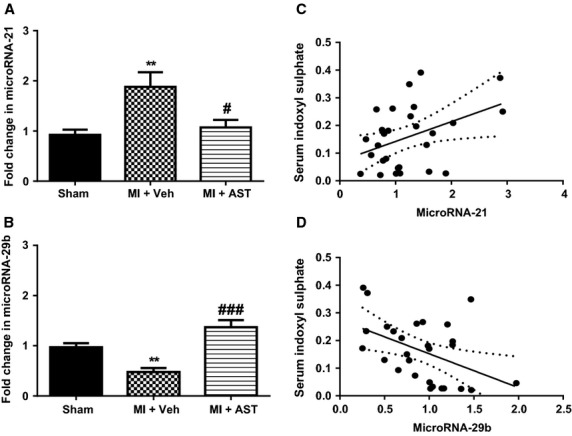
Quantitative analysis of (A) microRNA-21 and (B) microRNA-29b mRNA from sham (*N* = 12), MI+Vehicle (*N* = 8) and MI+AST-120 (*N* = 9) group of rats. Data presented as mean ± SEM. **P < 0.01 versus Sham. ^#^*P* < 0.05, ^###^*P* < 0.001 versus MI+Vehicle. Data analyzed by performing a one-way ANOVA followed by Bonferroni post hoc tests. (C) microRNA-21 correlated with Indoxyl Sulfate (*r*^2^ = 0.16; *P *=* *0.031; two-tailed Pearson's correlation test). (D) microRNA-29b correlated with Indoxyl Sulfate (*r*^2^ = 0.20; *P *=* *0.014; two-tailed Pearson's correlation test).

There was twofold increase in microRNA-21 mRNA expression in the noninfarct myocardium of the MI+Veh group compared to sham animals (*P* < 0.01, Fig.2A). MicroRNA-21 mRNA expression level in MI+AST-120 group was significantly lower compared to the MI+Veh group (*P* < 0.05, Fig.2A).

#### microRNA-29b

MicroRNA-29b mRNA expression in noninfarct myocardium was reduced by 50% in the MI+Veh group compared to sham animals (*P* < 0.01, Fig.2B). MicroRNA-29b mRNA expression level in MI+AST-120 was significantly higher compared to the MI+Veh group (*P* < 0.001, Fig.2B). There was no significant difference between levels of microRNA-29b mRNA in the sham and MI+AST-120 group (Fig.2B).

### Correlation between microRNA and IS

MicroRNA-21 mRNA levels were positively correlated with serum IS levels in rats (*P *=* *0.031; *r*^2^ = 0.16, Fig.2C). In contrast to microRNA-21, the microRNA-29b mRNA levels were negatively correlated with serum IS (*r*^2^ = 0.20; *P *=* *0.014, Fig.2D).

### Cardiac mRNA for extracellular matrix proteins and TGF- *β*1

**Figure 3:**
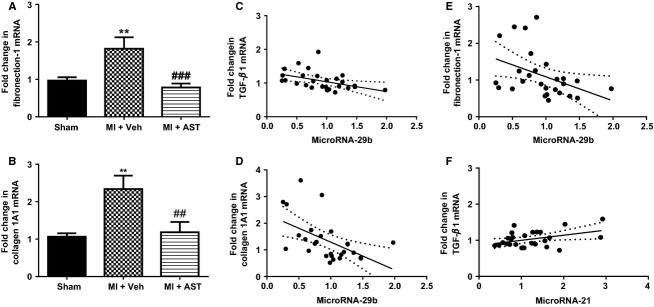
Quantitative analysis of mRNA for (A) collagen 1A1 and (B) fibronectin-1, from Sham (*N* = 12), MI+Vehicle (*N* = 8) and MI+AST (*N* = 9) group of rats. Data presented as mean ± SEM. **P < 0.01 versus Sham. ^##^*P* < 0.01, ^###^*P* < 0.001 versus MI+Vehicle. Data analyzed by performing a one-way ANOVA followed by Bonferroni post hoc tests. (C) MicroRNA-29b correlated with its target gene TGF-*β*1 (*r*^2^ = 0.19; *P *=* *0.019; two-tailed Pearson's correlation test). (D) MicroRNA-29b correlated with its target gene collagen 1A1 (*r*^2^ = 0.27; *P *=* *0.005; two-tailed Pearson's correlation test) (E) microRNA-29b correlated with its target gene fibronectin-1 (*r*^2^ = 0.17; *P *=* *0.03; two-tailed Pearson's correlation test) (F) MicroRNA-21 correlated with TGF-*β*1 (*r*^2^ = 0.17; *P* = 0.032; two-tailed Pearson's correlation test).

Compared to sham, cardiac mRNA levels for extracellular matrix proteins fibronectin-1 (*P* < 0.01, Fig.3A) and collagen 1 A1 (*P* < 0.01, Fig.3B) were higher in the MI+Veh group. This increase was significantly attenuated by AST-120 treatment (Fig.3A and B). TGF-*β*1 levels were 37% higher in MI+Veh group compared to sham (*P* < 0.01, Table1). TGF-*β*1 levels were 51% lower in the MI+AST-120 group as compared to the MI+Veh group (*P* < 0.001, Table1). There was no significant difference between levels of TGF-*β*1 mRNA in sham and MI+AST-120 groups.

### Cardiac mRNA for Spry-1, PTEN and Smad 7

**Figure 4:**
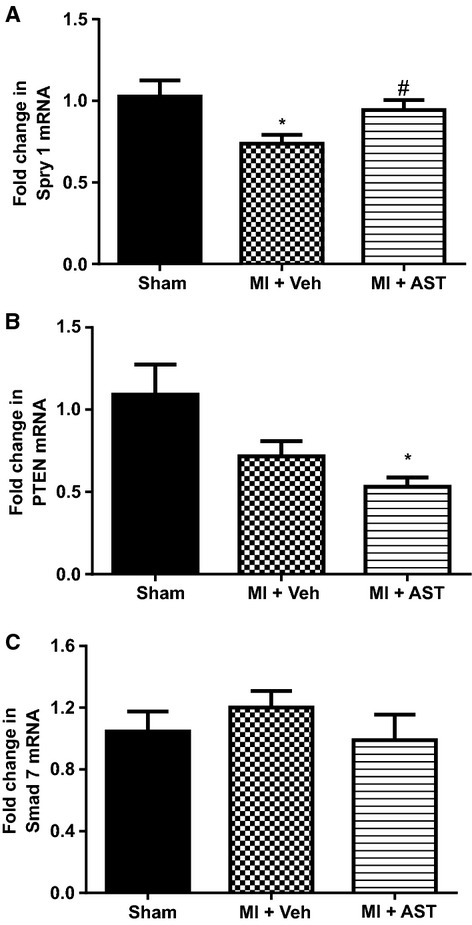
Quantitative analysis of mRNA for (A) Spry 1, (B) PTEN, and (C) Smad 7, from Sham (*N* = 12), MI+Vehicle (*N* = 8) and MI+AST (*N* = 9) group of rats. Data presented as mean ± SEM. *P < 0.05 versus Sham. ^#^*P* < 0.05 versus MI+Vehicle. Data analyzed by performing a one-way ANOVA followed by Bonferroni post hoc tests.

Compared to sham animals, Spry 1 mRNA expression was 27% lower in MI+Veh group of rats (*P* < 0.05, Fig.4A). AST-120 treatment completely restored Spry 1 expression to similar levels observed in sham rats (*P* < 0.05 vs. MI+Veh, Fig.4A). Compared to sham PTEN mRNA expression was 29% lower in MI+Veh group but did not reach significance (*P* = 0.09, Fig.4A). Compared to sham, PTEN levels were 47% lower in MI+AST-120 group of rats (*P* < 0.05, Fig.4B), but they were not significantly different from the MI+Veh group. Cardiac Smad 7 mRNA expression was not different between the three groups of animals (Fig.4C).

### Correlation between microRNA and target genes

Cardiac microRNA-29b mRNA level was negatively correlated with its target gene TGF-*β*1 (*r*^2^ = 0.19; *P* = 0.019, Fig.3C). MicroRNA-29b levels were negatively and significantly correlated with its target genes collagen 1A1 (*r*^2^ = 0.27; *P* = 0.005, Fig.3D) and fibronectin-1 (*r*^2^ = 0.17; *P* = 0.03, Fig.3E). Also, cardiac microRNA-21 mRNA level was positively correlated with cardiac TGF-*β*1 (*r*^2^ = 0.17; *P* = 0.032; Fig.3F).

### Cardiac collagen 1 protein levels

Cardiac collagen 1 protein levels were increased sixfold in vehicle-treated MI rats compared with sham animals (*P* < 0.001, Table1). This increase in MI-induced collagen 1 protein was completely attenuated by AST-120 treatment (*P* < 0.001 compared with MI+Veh, Table1). Collagen 1 protein levels in AST-120-treated MI rats were not different to sham rats. There was a significant positive correlation between collagen I protein levels and collagen 1A1 mRNA (*r*^2^ = 0.25; *P* = 0.018, Data not shown).

### Cardiac ACE and Agtr1a mRNA expression

**Figure 5:**
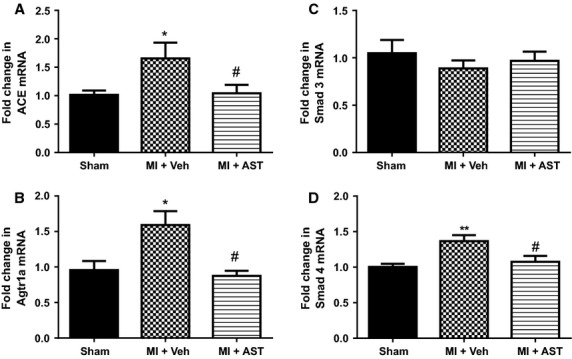
Quantitative analysis of mRNA for (A) ACE and (B) Agtr1a, from Sham (*N* = 12), MI+Vehicle (*N* = 8) and MI+AST (*N* = 9) group of rats. (C, D): Quantitative analysis of mRNA for Smad 3 and Smad 4, from sham (*N* = 12), MI+Vehicle (*N* = 8) and MI+AST (*N* = 9) group of rats. Data presented as mean±SEM. *P < 0.05, **P < 0.01 versus Sham. ^#^*P* < 0.05 versus MI+Vehicle. Data analyzed by performing a one-way ANOVA followed by Bonferroni post hoc tests.

Compared to sham animals, cardiac mRNA levels for ACE were 65% higher in the MI+Veh group (*P* < 0.05, Fig.5A). Agtr1a mRNA levels were 59% higher in MI+Veh group when compared with sham animals (*P* < 0.05, Fig.5B). These increases in ACE and Agtr1a mRNA expression were significantly attenuated by AST-120 treatment (Fig.5A and B).

### Cardiac Smad 3 and Smad 4 mRNA expression

Cardiac Smad 3 levels were not different between any of the three groups of animals studied (Fig.5C). Compared to the sham group, there was 36% increase in cardiac Smad 4 mRNA levels (*P* < 0.01, Fig.5D). This increase in Smad 4 expression was significantly attenuated by AST-120 treatment (*P* < 0.05, Fig.5D).

### Direct IS activation of microRNA and profibrotic signaling in primary cardiac fibroblasts

**Figure 6:**
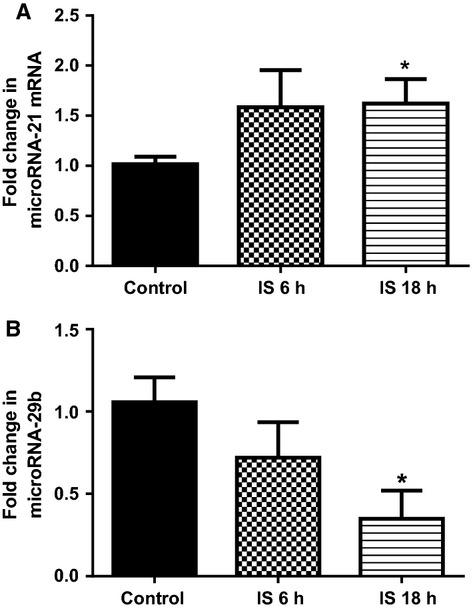
Quantitative analysis of (A) microRNA-21 and (B) microRNA-29b mRNA from cultured primary rat cardiac fibroblast cells following Control treatment (*N* = 3), and Indoxyl Sulfate treatment for 6 h (*N* = 3) and 18 h (*N* = 3). Data presented as mean ± SEM. *P < 0.05 versus Sham. Data analyzed by performing a one-way ANOVA followed by Bonferroni post hoc tests.

After 18 h of IS treatment, cardiac fibroblasts demonstrated a 60% increase in microRNA-21 mRNA expression, and a 68% decreased expression of microRNA-29b mRNA (*P* < 0.05 for both, compared to control, Fig.6A and B). Similar trends were observed following 6 h of IS treatment but these were not significantly different from control (Fig.6A and B).

**Figure 7:**
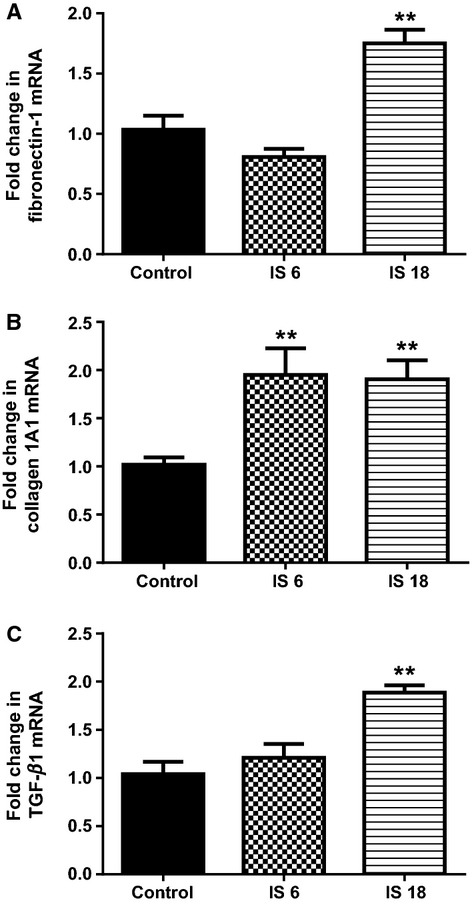
Quantitative analysis of mRNA for (A) fibronectin-1, (B) collagen 1A1 and (C) TGF-*β*1 from cultured primary rat cardiac fibroblast cells followingControl treatment (*N* = 3), and Indoxyl Sulfate treated for 6 h (*N* = 3) and 18 h (*N* = 3). **P < 0.01 versus Sham. Data analyzed by performing a one-way ANOVA followed by Bonferroni post hoc tests.

**Figure 8:**
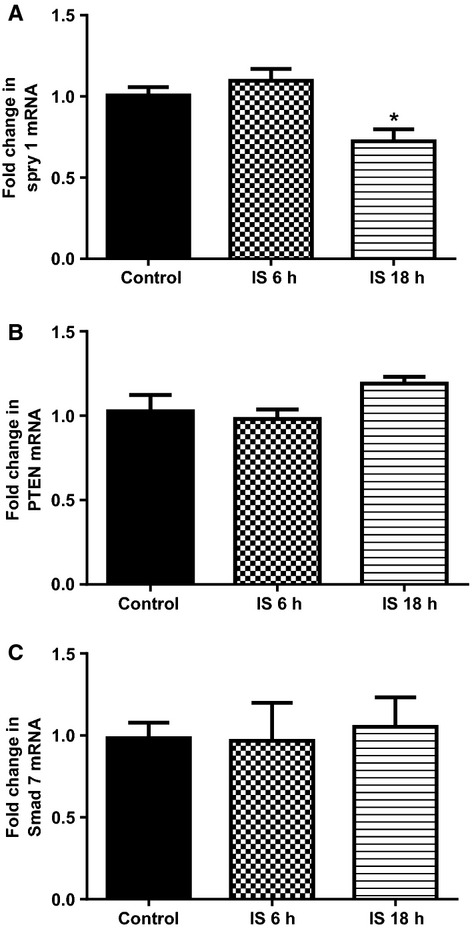
Quantitative analysis of mRNA for (A) Spry 1, (B) PTEN and (C) Smad 7 from cultured primary rat cardiac fibroblast cells following Control treatment (*N* = 3), and Indoxyl Sulfate treated for 6 h (*N* = 3) 18 h (*N* = 3). Data presented as mean ± SEM. *P < 0.05 versus control. Data analyzed by performing a one-way ANOVA followed by Bonferroni post hoc tests.

**Figure 9:**
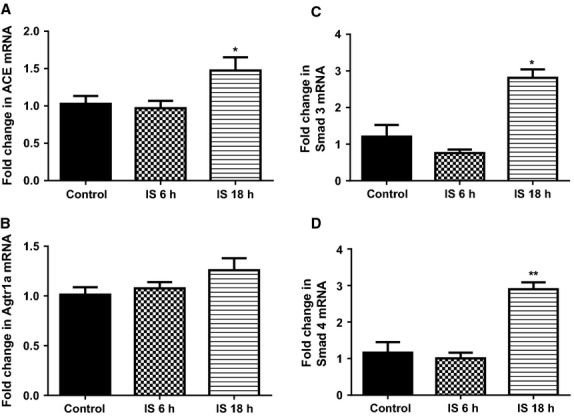
Quantitative analysis of mRNA for (A) ACE, (B) Agtr1a, (C) Smad 3, and (D) Smad 4 from cultured primary rat cardiac fibroblast cells following Control treatment (*N* = 3), and Indoxyl Sulfate treated for 6 h (*N* = 3) and 18 h (*N* = 3). Data presented as mean ± SEM. *P < 0.05, **P < 0.01 versus control. Data analyzed by performing a one-way ANOVA followed by Bonferroni post hoc tests.

mRNA expression of all three profibrotic genes fibronectin-1, collagen 1A1, and TGF-*β*1 were significantly higher after 18 h of IS treatment (*P* < 0.01 for all three genes, compared to control, Fig.7A–C). Only collagen 1 A1 had significantly higher mRNA expression as early as 6 h after IS treatment (*P* < 0.01, compared to control, Fig.7B). Expression of Spry 1 was significantly lower in cells treated with IS for 18 h (*P* < 0.05, Fig.8A). PTEN and Smad 7 mRNA expression were not different to control at any of the treatment times (Fig.8B and C). Expression of ACE was significantly higher in cells treated with IS for 18 h (*P* < 0.05, Fig.9A). Agtr1a mRNA expression was 25% higher after 18 h of IS treatment, however, this did not attain significance (Fig.9B). Compared to control, both Smad 3 and Smad 4 mRNA were significantly higher at 18 h after IS treatment (*P* < 0.05 for Smad 3 and *P* < 0.01 for Smad 4, Fig.9C and D).

## Discussion

This is the first study to report the regulation of cardiac microRNAs (21 and 29b) together with inhibition of fibrosis in the myocardium of MI rats after lowering uremic toxins levels. We report that exposure to elevated serum IS causes an increase in microRNA-21 mRNA expression along with reduced mRNA expression of microRNA-29b in the heart. We report a significant correlation between cardiac microRNA-21 and serum IS, and a significant but inverse association between cardiac microRNA-29b and serum IS. These two microRNAs govern cardiac fibrosis through their ability to regulate the fibrotic actions of TGF-*β*1, a key mediator of cardiac fibrosis. Furthermore, activation of the angiotensin system was observed in vivo with increased gene expression of Agtr1a and ACE. Treatment with AST-120 inhibited cardiac fibrosis, ACE, Agtr1a, Smad 4, microRNA-21 expression and restored cardiac microRNA-29b mRNA expression toward Sham levels suggesting activation of the cardiac renin-angiotensin system as a mechanism for altering microRNAs expression by uremic toxins such as IS.

Expression of microRNAs (21 and 29b) in the heart post-MI has been previously determined. van Rooij et al. (2008) reported reduced microRNA-29b in peri-infarct and remote myocardium within 3 days of MI induction, which was still significantly lower in peri-infarct but not in remote myocardium at 2 weeks post-MI. Earlier studies in rodent models have reported altered expression of microRNA-21 within the first 2 weeks after induction of MI (Dong et al. 2009; Liang et al. 2012).

Neonatal cardiac cells were used in this study since they are a proven model of cardiac fibrosis and are useful for evaluating the efficacy of therapeutic agents (Thomas et al. 2002a,b; Woodcock et al. 2002) Neonatal cardiac cells also express the fetal gene program which is reexpressed in adult cells and tissues with heart failure (Woodcock et al. 2002; Taegtmeyer et al. 2010). Given that chronic left ventricular dysfunction, in an MI model is known to cause pathological fibrosis with regulatory effects on miRNAs, we sought to determine whether IS, a profibrotic molecule, could regulate these miRNAs in cardiac fibroblast culture. Our fibroblasts data with IS treatment demonstrate a clear role for IS in altering expression of these microRNAs and their target genes mimicking the in vivo data. The in vitro cardiac fibroblast data showed increased collagen IA1 following IS stimulation but not fibronectin or TGF*β*_1_ gene expression at the earlier time point of 6 h, this may be due to the effects of IS on pathways other than TGF*β*_1_. We have previously reported IS-mediated increases in nuclear factor kappaB activation in neonatal cardiac fibroblasts (Lekawanvijit et al. 2010) which may be a result of increased reactive oxygen species that induces collagen synthesis. Subsequently IS may induce fibrotic genes such as TGF*β*_1_ and fibronectin at the later time point investigated in this study. Further investigation over several time points needs to be performed to clarify the involvement of these pathways.

The dose of IS in our cell culture experiments is equivalent to the concentration of IS measured in the serum of vehicle-treated MI animals at 16 weeks. Our animal data suggest that AST-120 treatment prevented uremic toxin-induced attenuation of cardiac microRNAs-29b post-MI, leading to significant downregulation of microRNA-29b target genes TGF-*β*1, collagen 1A1 and fibronectin-1. Studies have shown that overexpression of microRNA-29b in cardiac fibroblasts prevents the angiotensin II-mediated increase in collagen 1 mRNA and protein (Zhang et al. 2014). MicroRNA-29 overexpression and knockdown studies in cardiac cells and other cell types have reported its inhibitory action on the TGF-*β*1 pathway (Cushing et al. 2011; Luna et al. 2011; Roderburg et al. 2011; Zhang et al. 2014). MicroRNA-29b directly binds to exon 3 coding sequence for TGF-*β*1 to promote its mRNA degradation and inhibit its translation (Zhang et al. 2014). TGF-*β*1 can also inhibit microRNA-29b expression by activating the Smad signaling pathway (Qin et al. 2011; Winbanks et al. 2011; Xiao et al. 2012; Zhang et al. 2014). Collectively, these findings suggest the presence of a negative reciprocal loop between TGF-*β*1 and microRNA-29b, where activation of one leads to inhibition of the other. Our results suggest that uremic toxins modulate this loop favoring inhibition of microRNA-29b and promoting cardiac fibrosis.

The profibrotic cytokine TGF-*β*1 is a key regulator of fibrosis in cardiac fibroblasts (Youn et al. 1999; Bujak and Frangogiannis 2007). Fibrotic pathways activated by TGF-*β*1 involve increased cardiac synthesis of ECM proteins fibronectin and collagens (I-III) (Eghbali et al. 1991; Heimer et al. 1995; Villarreal et al. 1996), their accumulation in the myocardium has been reported following MI (Seeland et al. 2002; van Dijk et al. 2008; Dworak et al. 2012; Lekawanvijit et al. 2013). Excessive collagen and ECM protein deposition in the heart are known to cause arrhythmia, mechanical stiffness, disorganized contraction, and hypoxia, which ultimately contribute to the development of heart failure. Studies on pathologies of the heart, kidney, and lung involving fibrosis have reported altered expression of microRNA-21 and microRNA-29b along with increased expression of TGF-*β*1 (Thum et al. 2008; Cushing et al. 2011; Qin et al. 2011; Roderburg et al. 2011; Noetel et al. 2012; Patel and Noureddine 2012; Xiao et al. 2012; Wang et al. 2013a,b).

It is well established that the TGF-*β*1 signaling pathway is regulated by intracellular signaling proteins known as Smads which play an important role in fibrosis. Increased protein levels of Smad 2, 3, and 4 have been reported in cardiac scar and border tissue collected from rats 8 weeks after MI (Hao et al. 1999). TGF-*β*1 binds to its cell surface receptors and phosphorylates Smad 2 and Smad 3 (also known as R-Smad) which form a bond with Smad 4 (also known as co-Smad) in the cytoplasm. This complex translocates to the nucleus where it binds to the promoter region of microRNAs and TGF-*β*1 target genes to modulate their expression (Tang and Lan 2014). Our results show increased cardiac mRNA expression of Smad 4 but not Smad 3 in vehicle-treated MI rats. Inhibition of this increase in Smad 4 by AST-120 treatment, and upregulation Smad 3 and Smad 4 expression in IS-treated cardiac fibroblast cells, suggest Smads are involved in IS-mediated alteration of cardiac microRNAs and profibrotic gene expression. The role of Smads in the kidney has been well documented in renal fibrosis which may help explain our findings in the heart. Smad3-deficient mice are protected from renal tubulointerstitial fibrosis in the unilateral ureteral obstruction model (Zhong et al. 2011), and inhibition of Smad4 expression by siRNAs in mice significantly inhibited renal fibrosis induced by folic acid (Morishita et al. 2014). A recent study using Smad 4 knockout mice has reported attenuation of renal fibrosis in a unilateral ureteral obstruction model (Meng et al. 2012). Thus, our data indicate that the inhibition of Smad 4 mRNA by lowering serum IS levels, appears to be a mechanism for the inhibition of fibrosis.

Angiotensin II is an important neurohormone that also plays a pathogenic role in cardiac remodeling post-MI, and is known to stimulate the synthesis and secretion of TGF-*β*1 (Sharma et al. 1994; Yang et al. 2009) and activate the Smad pathway (Rodriguez-Vita et al. 2005) (Sun et al. 1998) via either or both, TGF-*β*1 and/or p38/extracellular signal-regulated kinase/mitogen-activated protein kinase (p38/ERK/MAPK) pathways (Rodriguez-Vita et al. 2005; Yang et al. 2009). Our results showing increased cardiac ACE expression after MI and IS treatment in cardiac fibroblasts indicate that IS may upregulate local angiotensin II signaling in cardiac cells to increase TGF-*β*1 expression. Angiotensin II is already known to upregulate TGF-*β*1 expression via activation of the Agtr1 receptors in cardiac cells mediating cardiac fibrosis (Rosenkranz 2004; Dobaczewski et al. 2011). The Agtr1 receptor antagonist losartan has been shown to inhibit protein expression of both Smad 4 and the active form of TGF-*β*1 in scar tissue from MI rats (Hao et al. 2000). Therefore, our results demonstrating inhibition of Agtr1a expression in AST-120-treated rats, compared with vehicle MI rats, suggest that activation of Agtr1a receptors by uremic toxins may be responsible for the increase in Smad 4 mRNA expression.

Angiotensin II also has been reported to regulate expression of microRNA-29 via Smads. Chronic angiotensin II infusion in wild-type mice resulted in significant reduction in microRNA-29 while in Smad3 knockout mice this reduction was prevented (Zhang et al. 2014). In addition, overexpression of microRNA-29b in mice prevented angiotensin II-induced cardiac fibrosis, and inhibited TGF-*β*1, Smad 3 activation, and reduced phosphorylation of ERK1/2 kinases. Furthermore, the increased Agtr1a and decreased microRNA29b post-MI and their reversal following AST-120 treatment may involve elements of R-Smad.

Increased microRNA 21 expression in cardiac fibroblasts from failing heart has previously been reported, and this augments ERK-MAP kinase activity through inhibition of Spry1 ^8^. In addition, angiotensin II treatment of cardiac fibroblasts has previously been reported to increase microRNA-21 expression and decrease Spry1 expression (Adam et al. 2012). Here, we report a significant reduction in the cardiac expression of microRNA-21's target gene Spry1 in vehicle-treated MI rats, and in IS-treated cardiac fibroblast cells. In vivo silencing of microRNA-21 in a pressure-overload model reduced cardiac ERK-MAP kinase activity, inhibited interstitial fibrosis and attenuated cardiac dysfunction (Thum et al. 2008). PTEN and SMAD 7 are also direct targets for microRNA-21, and an increase in microRNA-21 expression has an inhibitory effect on their expression. IS treatment in cardiac fibroblasts did not have any effect on mRNA expression of these genes despite significant increases in expression of microRNA-21. Although, we did observe a nonsignificant reduction in PTEN mRNA expression in vehicle-treated MI rats, AST-120 treatment did not restore their expression, suggesting involvement of mechanisms independent of uremic toxins.

AST-120 reduced cardiac fibrosis in MI animals without an improvement in cardiac function. Fibrosis in the noninfarcted region, although increased in vehicle-treated animals is still small in comparison to the injured infarcted area which is normally 10 times greater. Hence a small reversal of fibrosis (<2% Fig.1) in the noninfarcted region may not significantly contribute to the overall function of the heart. Additionally a period longer than 16 weeks post-MI may be required to see improvements in functional outcomes as the function deteriorates further in MI+Veh-treated rats more than that of the AST-120-treated rats.

We opted not to administer uremic toxins into nonoperated animals, since their normal renal function would likely cause these toxins to be excreted in urine without impacting on the kidney or the heart. Studies have shown that in models of significantly impaired renal function, have demonstrated using a chronic renal injury model, addition of uremic toxins such as indoxyl sulfate results in addition worsening of glomerulosclerosis, fibrosis, and reduced function (Niwa and Ise 1994; Niwa 2013).

**Figure 10:**
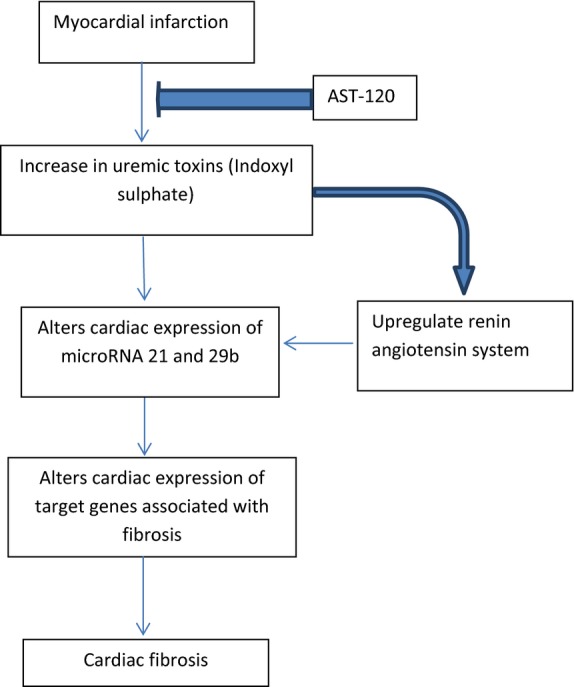
Diagram outlines hypothesis and key findings of this study.

Measurement of Smad 3 and 4 phosphorylation levels may provide more information in future studies regarding the regulation of microRNA 21 and 29b-mediated fibrosis. In summary, as outlined in Fig.10 our study provides evidence that elevated IS levels caused by renal damage secondary to MI has a direct effect of on the expression of microRNA-21 and microRNA-29. This altered cardiac microRNA expression after MI in turn increases the expression of TGF-*β*1 and leads to cardiac fibrosis in the myocardium. These findings suggest that treatments targeting microRNA-21 and microRNA-29b may be a potential therapy for fibrosis in MI patients with concomitant chronic kidney disease.
