# Contribution of microRNA to pathological fibrosis in cardio-renal syndrome: impact of uremic toxins

**DOI:** 10.14814/phy2.12452

**Published:** 2015-06-24

**Authors:** Indrajeetsinh Rana, Andrew R. Kompa, Joanna Skommer, Bing H. Wang, Suree Lekawanvijit, Darren J. Kelly, Henry Krum, Fadi J. Charchar

In Rana et al. (2015), the following errors were published in the Results, Discussion and Figures 2 and 6:

The authors used the terms “microRNA-21 mRNA” and “microRNA-29b mRNA,” which are scientifically incorrect. The correct terms are “microRNA-21” and “microRNA-29b.”

The authors apologise for the errors.
